# A comparison of extracorporeal and conventional cardiopulmonary resuscitation for cardiac arrest

**DOI:** 10.1186/s13054-024-04872-9

**Published:** 2024-03-18

**Authors:** Yang Zhao, Qian Wang, Bin Zang

**Affiliations:** 1 Department of Critical Care Medicine, Shengjing Hospital of China Medical University, Shenyang, 110000 China; 2 Department of Emergency, The Fourth Affiliated Hospital of China Medical University, Shenyang, 110000 China

To the Editor,

We are highly interested in the recent article published in *Critical Care* by Low CJW et al., titled "Extracorporeal cardiopulmonary resuscitation versus conventional CPR in cardiac arrest: an updated meta-analysis and trial sequential analysis" [1]. In updating their previous systematic review and meta-analysis [2], the authors found that extracorporeal cardiopulmonary resuscitation (ECPR) reduces in-hospital mortality compared to conventional cardiopulmonary resuscitation (CPR) and indicated the potential for ECPR application in both in-hospital and out-of-hospital cardiac arrest (OHCA).

In this meta-analysis, the authors focused mainly on updating mortality rate data for patients with OHCA, placing less emphasis on in-hospital cardiac arrest (IHCA) patients. Using the authors' search strategy, we identified a new study that compares ECPR with CCPR in IHCA patients via a propensity score matching cohort study [3]. After incorporating this study, we performed a meta-analysis with Stata version 16.0, concentrating on the mortality of IHCA patients. The meta-analysis results indicated a significant reduction in in-hospital mortality for IHCA patients with ECPR (RR, 0.83; 95% CI 0.75–0.91, *P* < 0.05) (Fig. 1).

**Fig. 1:**
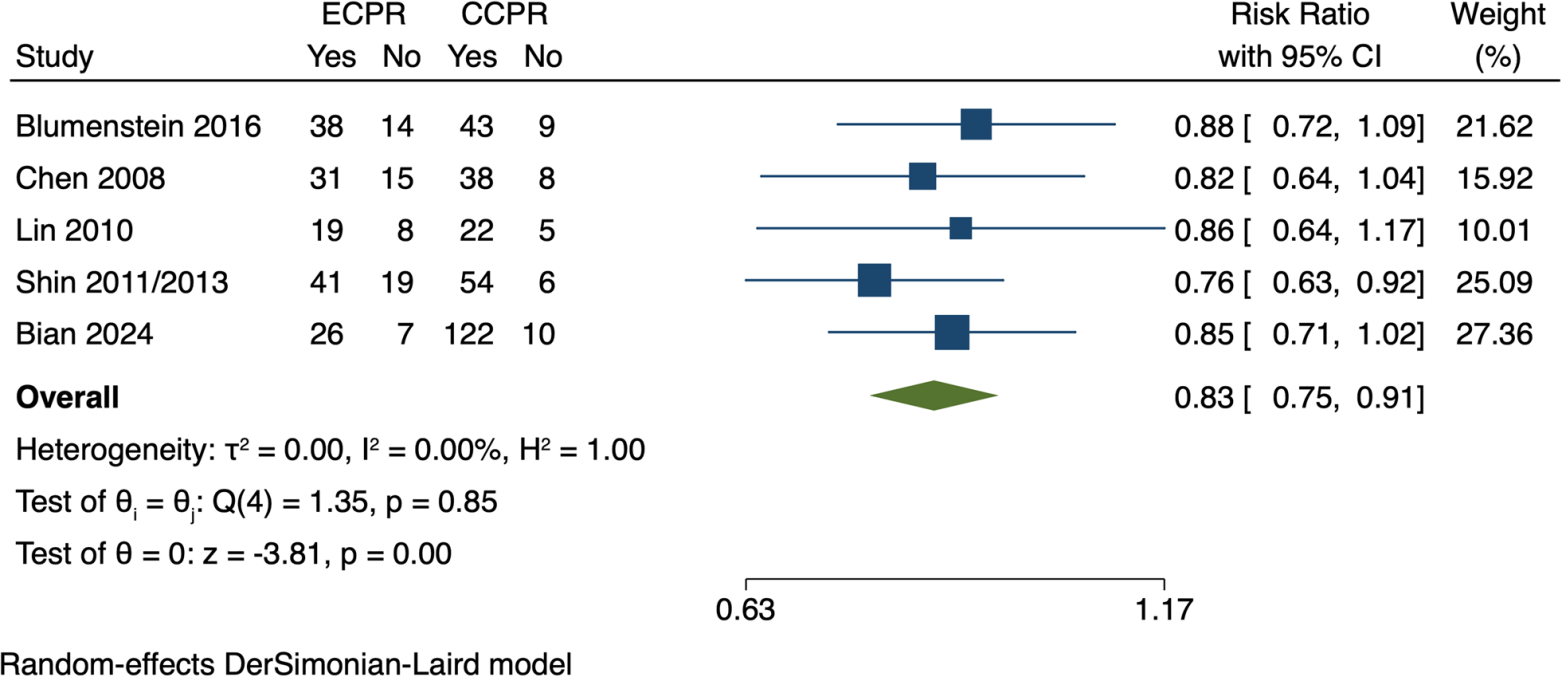
Forest plot of mortality in IHCA patients. ECPR, extracorporeal cardiopulmonary resuscitation; CCPR, conventional cardiopulmonary resuscitation; IHCA, in-hospital cardiac arrest; CI, confidence interval

We conducted a trial sequential analysis (TSA) using TSA viewer version 0.9.5.10 Beta to assess the reliability of the results from the meta-analysis and the risk of type I (false-positive) and type II (false-negative) errors. The results showed that the Z-curve crossed both the conventional boundary and the required information size, yet it did not cross the TSA boundary (Fig. 2). This suggests that the current sample size might be insufficient for reliable conclusions. Consequently, the observed survival benefit of ECPR compared to CCPR for IHCA patients may potentially be a false-positive finding. Thus, further research is required to validate this outcome.

**Fig. 2:**
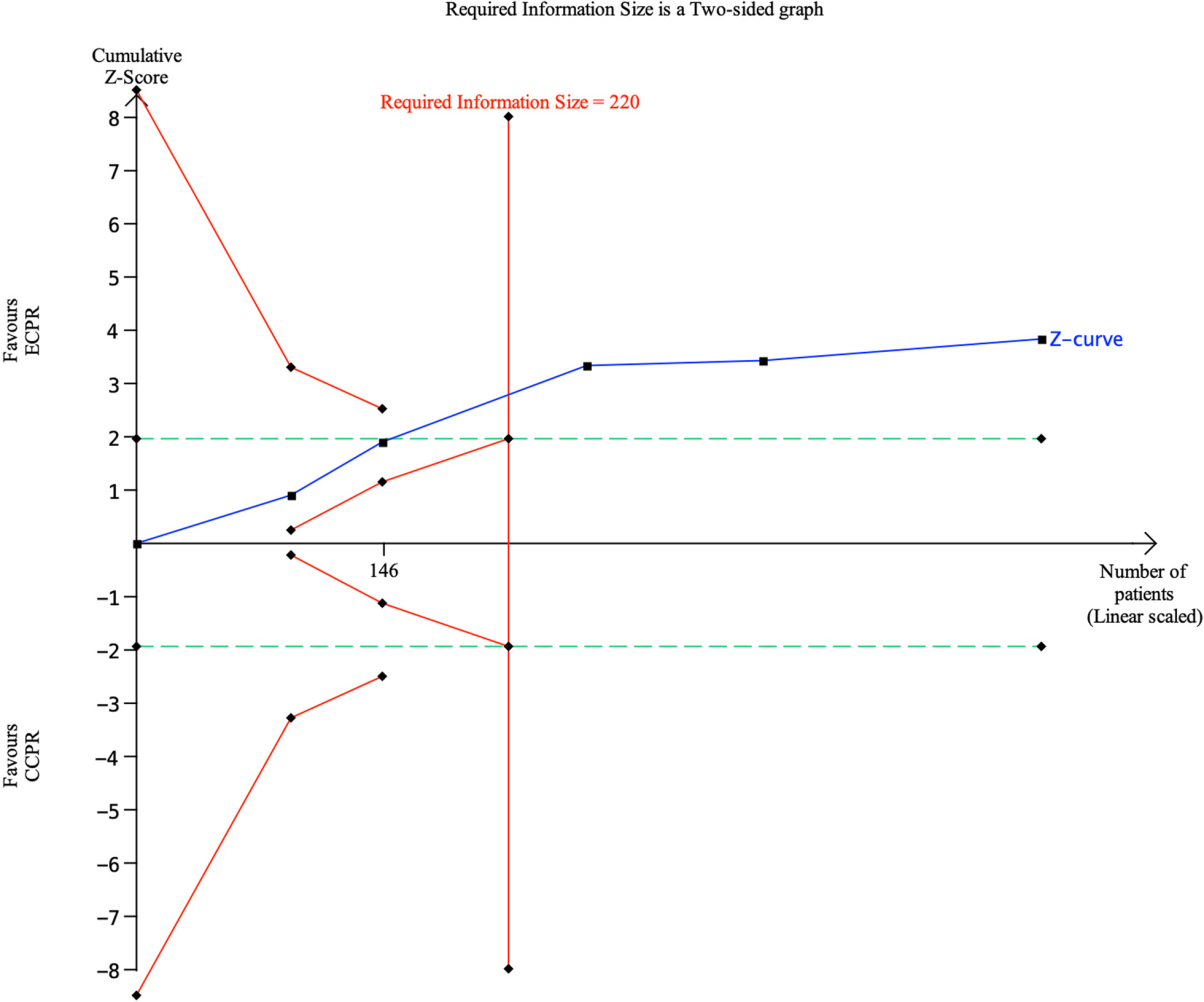
Trial sequential analysis of mortality in IHCA patients. The blue Z curve represents the treatment effect (pooled relative risk). Green dotted lines denote conventional boundaries, and red solid lines indicate TSA boundaries. ECPR, extracorporeal cardiopulmonary resuscitation; CCPR, conventional cardiopulmonary resuscitation; IHCA, in-hospital cardiac arrest

Furthermore, in Low CJW's Additional File 1: Table S3, concerning overall mortality and 30-day survival, the Z-curve failed to surpass the TSA boundary despite meeting the required information size. This outcome implies that although cumulative evidence indicates statistical significance in traditional analysis, from the perspective of TSA, this significance may be due to random error. Therefore, the current evidence might not sufficiently establish the efficacy of ECPR for cardiac arrest, necessitating further studies for confirmation.

## Data Availability

Not applicable.
